# A Multivariate Analysis of Depression Prevalence in Psoriasis Patients: A Cohort Study

**DOI:** 10.3390/ijerph19042060

**Published:** 2022-02-12

**Authors:** Michele Fabrazzo, Francesca Romano, Marzia Arrigo, Rosa Valentina Puca, Antonietta Fuschillo, Valeria De Santis, Gaia Sampogna, Giulia Maria Giordano, Francesco Catapano, Ada Lo Schiavo

**Affiliations:** 1 Department of Psychiatry, University of Campania “Luigi Vanvitelli”, Largo Madonna delle Grazie 1, 80138 Naples, Italy; marziarrigo@hotmail.com (M.A.); ainot@libero.it (A.F.); valedesa1984@libero.it (V.D.S.); gaia.sampogna@gmail.com (G.S.); giuliamgiordano@gmail.com (G.M.G.); francesco.catapano@unicampania.it (F.C.); 2 Dermatology Unit, University of Campania Luigi Vanvitelli, 80131 Naples, Italy; dott.francescaromano@gmail.it (F.R.); dott.puca@gmail.com (R.V.P.); ada.loschiavo@unicampania.it (A.L.S.)

**Keywords:** coping strategies, depression, mental health assessment tools, mood disorders, psoriasis, psychological distress

## Abstract

The literature reported higher depression rates in psoriasis patients compared to the general population. Our study aimed to verify whether variability in depression prevalence was due to using different diagnostic tools. We also aimed to determine whether dysfunctional coping strategies might increase the depression burden. We assessed psoriasis severity by the Psoriasis Area Severity Index (PASI) and PSOdisk. We analyzed mental alterations of 120 outpatients by Hamilton Depression and Anxiety Rating Scales (HAM-D and HAM-A), Symptom Checklist-90-Revised (SCL-90-R), plus coping strategies and quality of life by Coping Orientation to Problems Experienced (COPE) Inventory and 36-Item Short Form Health Survey (SF-36). We divided our cohort into five subgroups from minimal to severe psoriasis using the PSOdisk total score. Depression prevalence varied according to the assessment criteria for specificity, frequency, and severity. Different mood disorders other than major depression emerged when we used DSM-IV-TR criteria. Correlation analysis of the criteria we used to diagnose depression or depressed mood indicated that a dysfunctional coping strategy was highly and positively correlated only in patients of the severe subgroup. Differently, a negative correlation emerged between the SF-36 Mental Summary Component (MSC) and behavioral disengagement, thus suggesting that psychopathological distress might induce patients with a marked/severe psoriasis to adopt dysfunctional coping strategies. Dermatologists are fundamental in detecting comorbid depression, referring psoriasis patients to mental health specialists to achieve adequate treatments, and preventing suicide risk.

## 1. Introduction

Previous studies have reported a higher incidence of depression in psoriasis patients compared to the general population [1,2,3,4,5,6,7]. The percentage of psoriasis patients with depressive symptoms ranges from 9% to 55%, likely due to different assessment criteria and study populations [8]. A recent cross-sectional study reported an incidence of 13.9% for moderate-to-severe depression in patients with psoriasis vulgaris [9]. Comorbid depression may also represent an essential indicator of psychological distress in psoriasis patients and a determinant of poor health-related quality of life (QoL) that causes reduced functioning, cognitive impairment, and increases social distress [10,11,12,13]. Psoriasis patients often feel limited by their physical appearance and inclined to isolate themselves, avoiding social interactions [14]. This is a result of increased itch, social stigmatization, joint arthropathic and clinical manifestations, in addition to poor treatment adherence, may contribute to mood severity. Such components, alone or in combination, may add anxiety, stress-related disorders, and even suicidal ideation to depression [15,16].

Schmitt and Ford [17] hypothesized that everyday life adversities and inadequate coping strategies might cause depression worsened by an impaired QoL. However, psoriasis patients might present other comorbidities liable to increase the risk of developing depression (i.e., cardiovascular diseases, obesity, diabetes, hypertension, dyslipidemia, and metabolic syndrome) [18,19,20].

Recognizing mood disorders may prove troublesome for non-psychiatric physicians with the consequent delay of the illness outcome and administration of the appropriate therapy [21,22,23].

In addition, most dermatologic patients often do not contact mental health providers when significant psychological distress is clinically identified [24].

Only a few studies reported a depression diagnosis based on standardized psychiatric criteria and/or a face-to-face psychiatric interview in patients with psoriasis [4]. Indeed, most authors did not specify whether they diagnosed depressive disorders or merely recognized mood symptoms. Furthermore, several dermatologists underestimated the prevalence of psychiatric morbidity in their patients [25,26].

Therefore, detecting clinical and psychopathological depression symptoms in patients with psoriasis is crucial to ensure optimal care.

In the present study, we hypothesized that the comorbid depression severity parallels that of psoriasis in a cohort of dermatologic outpatients. We further aimed to prove that comorbid depression may present a different prevalence when using multivariate diagnostic criteria to assess the sole depressive mood symptom or depression as a clinical disorder.

Furthermore, we supposed that the comorbid depression burden might also be correlated with dysfunctional coping strategies adopted by psoriasis patients to manage the skin disease and the associated mental complications.

## 2. Materials and Methods

### 2.1. Subjects

We conducted our open-label study at the Unit of Dermatology in cooperation with the Department of Psychiatry of the University of Campania “Luigi Vanvitelli”, Naples, from December 2014 to April 2019. Experienced dermatologists of the Unit of Dermatology enrolled 120 outpatients and made the diagnosis of psoriasis.

Admission criteria were the following: (a) diagnosis of psoriasis scoring from mild to very severe according to the Psoriasis Area Severity Index (PASI), with a score ≥3); (b) previous treatments with topic and/or systemic dermatological drugs and a screening to commence biological therapy; (c) willingness to undertake a psychological assessment; (d) age 18–70 years; (e) sufficient comprehension of the Italian language.

Recruited patients provided written informed consent after receiving a full description of the study. In addition, an Institutional Board reviewed the study protocol (no. 130/2014), conducted according to the updated version of the Declaration of Helsinki for experiments involving humans.

At baseline, we used an anamnestic form to record patients’ features, which were: age, gender, marital and employment status, educational level (sociodemographic characteristics), age at psoriasis onset, illness duration and outcome, previous therapies, number of relapses, stressful life events at onset and relapses, lifestyle habits, family and personal history of psychiatric disorders (clinical and psychological characteristics).

Experienced psychiatrists evaluated the psychopathological condition of each patient and made the diagnosis of depression after face-to-face interviews based on the Diagnostic and Statistical Manual of Mental Disorders, Fourth Edition, Text Revision (DSM-IV-TR), and confirmed by the Structured Clinical Interview for DSM-IV, Axis I (SCID-I) [27].

### 2.2. Assessments

We administered to patients recruited the validated Italian version of the following instruments: the PASI, the PSOdisk, the Symptom Checklist-90-Revised (SCL-90-R), the Hamilton Depression Rating Scale (HAM-D), the Hamilton Rating Scale for Anxiety (HAM-A), the Short Form-36 Health Survey (SF-36), and the Coping Orientation to Problems Experienced (COPE) Inventory.

#### 2.2.1. Psoriasis Evaluation: PASI and PSOdisk

The PASI is a self-report measure of psoriasis severity, designed to enable the patient to evaluate the severity of his/her current psoriatic plaques [28]. The physician in charge assigns the patients’ ratings to the PASI intensity and extent scales and calculates intensity and extent of the psoriatic plaques for each of the four anatomical regions (head, trunk, upper and lower extremities).

The PASI score varies from 0 to 72, with higher scores indicating severer conditions [29].

PSOdisk is a 10-item questionnaire issued in Italian that assesses psoriasis burden on patients through an intuitive visual outcome of the test [30]. The questionnaire is designed to be completed jointly by patients and dermatologists. The answers are given on a 10-point visual analog scale, graphically represented on a disk as a polygon, and varying from “absolutely not” to “definitely yes”.

The PSOdisk ten subscales include the following items: (i) health, (ii) pain, (iii) itch, (iv) sleep, (v) social life, (vi) work and other daily activities, (vii) peace of mind, (viii) sexual life, (ix) shame, and (x) skin involvement. According to Sampogna et al. [31], we defined five categories of disease burden: (i) minimal (PSOdisk total score < 9); (ii) mild (9–15); (iii) moderate (16–30); (iv) marked (31–50); (v) severe (>50).

#### 2.2.2. Psychopathological Assessment

We assessed the severity of depression by the HAM-D, which contains 21 items evaluating somatic, cognitive, and emotional depressive symptoms. The score range is 0–52, with the following cut-off scores: 0–7 (absence of depression), 8–17 (mild depression), ≥18 (moderate/severe depression) [32].

We determined the anxiety level also through the HAM-A, a 14-item clinician-rated questionnaire measuring both psychic (mental agitation and psychological distress) and somatic anxiety (physical complaints). Each item is scored on a 5-point scale ranging from 0 (not present) to 4 (severe), with a total score between 0 and 56. The following total scores indicate different conditions of anxiety: mild (≤17), mild to moderate (18–24), and moderate to severe (25–30) [33].

We evaluated general psychopathology by the SCL-90-R checklist [34], a multidimensional self-report symptom inventory designed to screen and detect clinical symptoms or indicators of psychological distress. The checklist includes 90 items on a 5-point scale (from 1 = no problem to 5 = very serious) to measure the extent of the listed symptoms experienced in the last seven days. The items are divided into nine subscales: (i) somatization, (ii) obsessive-compulsive, (iii) interpersonal sensitivity, (iv) depression, (v) anxiety, (vi) anger-hostility, (vii) phobic anxiety, (viii) paranoid ideation, (ix) psychoticism. A high score on the SCL-90-R indicates great psychological distress. The SCL-90-R also includes three global indexes: the global severity index (GSI), which measures the extent or depth of the individual’s psychiatric disturbances; the Positive Symptom Total (PST), which counts the total number of questions rated above 1 point; and the Positive Symptoms Distress Index (PSDI), calculated by dividing the sum of all items values by the PST. In this study, we only reported GSI and raw scores of SCL-90-R subscales. Based on the study of Nojomi and Gharayee [35], we used the cut-off point of 0.7 for GSI. A score of 0.7 and above was considered as indicative of a possible case of mental disorder.

#### 2.2.3. Quality of Life and Coping Strategies Evaluation: SF-36 and COPE Inventory

The 36-Item Short Form Health Survey (SF-36) is a set of easily administered instruments for measuring health-related QoL or functional health status [36]. The 36 items refer to the last 4 weeks, except for the physical functioning (PF) scale (“at this moment”) and general health perceptions (GHP) (“in general”). These measures rely upon patient self-reporting and include 36 items grouped into the following eight dimensions: (i) vitality (VT), (ii) physical functioning (PF), (iii) bodily pain (BP), (iv) general health perceptions (GH), (v) role limitations due to physical problems (RP), (vi) role limitations due to emotional problems (RE), (vii) social functioning (SF), and (viii) general mental health (MH) [36,37]. In addition, scores for physical and mental health can be calculated by the SF-36 scales: the physical component summary (PCS) includes PF, RP, BP, and GH, while the mental component summary (MCS) includes role limitations caused by VT, SF, RE, and MH. The PCS and MCS are calculated by standardizing each dimension by means and standard deviations of the Italian population for its subsequent aggregation and transformation [37,38]. A higher score means better health, with a score of 0 indicating maximum disability, and of 100 no disability.

The Coping Orientation to Problems Experienced (COPE) Inventory [39] is a 60-item measure comprising 15 subscales with 4 items each, developed to assess the different coping strategies, problem- and emotion-focused, used in response to stress. Problem-focused coping is directed at problem-solving actions that comprise acting to alter the stressor. Emotion-focused coping responses, contrarily, are directed at reducing or managing the emotional distress produced by the situation.

#### 2.2.4. Statistical Analysis

As appropriate, we described sociodemographic and clinical variables, including the severity of psoriasis, duration of the illness, and psychiatric assessments by frequency counts, mean and standard deviations. Specifically, we used the PASI total score and PSOdisk total and subscale scores, the HAM-D depressed mood item mean score, the HAM-D, HAM-A, SCL-90-R, and GSI mean total scores, plus the COPE Inventory subscale scores.

Patients with psoriasis were divided into five subgroups according to the criteria reported by Sampogna et al. [31]. Differences in the primary clinical subscales among these subgroups were assessed using the one-way ANOVA test with post hoc Bonferroni correction.

Prevalence of depression was determined as follows: by HAM-D depressed mood item, HAM-D total score (≤7 = no depression, 8–17 = mild depression, and ≥18 = moderate/severe depression), SCL-90-R GSI score (≥0.70 = psychological distress), and DSM-IV-TR criteria for mood disorders. Differences among groups were evaluated using a chi-square test or one-way ANOVA test with post hoc Bonferroni correction.

Furthermore, differences in coping strategies among subgroups of patients with psoriasis were determined using the one-way ANOVA test with post hoc Bonferroni correction. Subsequently, a correlation analysis was performed to evaluate the relationship between the behavioral disengagement COPE subscale score and psychopathological diagnostic tools (HAM-D depressed mood item and SCL-90-R depression subscale scores, HAM-D, HAM-A, and SCL-90-R GSI total scores), in addition to the SF-36 Mental Health subscale score using Pearson’s correlation coefficient (r).

The Statistical Package for Social Sciences (SPSS^®^) version 18.0 was used, and the level of significance was set at *p* < 0.05.

## 3. Results

### 3.1. General Characteristics of the Enrolled Patients

Sociodemographic and clinical characteristics of the 120 patients enrolled (45 women and 75 men) are reported in Table 1. According to the PSOdisk total score, we divided our cohort into five subgroups: minimal (<9), mild (9–15), moderate (16–30), marked (31–50), and severe (>50). No significant differences emerged from the analysis of all the variables listed in Table 1, except for gender and occupational status (*p* < 0.0001 and *p <* 0.05) of the five subgroups patients.

In our cohort, 103 patients were diagnosed with psoriasis vulgaris. Specifically, 15 belonged to the minimal subgroup, four to the mild, six to the moderate, 24 to the marked, and 54 to the severe. In addition, one patient of the minimal and two patients of the severe subgroups were diagnosed with plaque psoriasis; one patient of the moderate subgroup with guttate, and one patient of the minimal subgroup with palmoplantar psoriasis.

A few patients were diagnosed with arthropathic psoriasis, which was the most relevant in the severe subgroup (Table 2).

The PASI total score was more elevated in psoriasis patients of the severe subgroup compared to those of the moderate subgroup (*p* < 0.05) (Table 2). Moreover, the PSOdisk subscale scores of itch (*p* < 0.0001), shame (*p* < 0.0001), and skin involvement (severe vs. minimal *p* < 0.0001, vs. mild *p* < 0.001, vs. moderate *p* < 0.009, and vs. marked *p* < 0.05) significantly differed when we compared severe subgroup patients to those of all the other subgroups.

### 3.2. Psychopathological Characteristics of Psoriasis Patients

The data analysis revealed that severe and marked subgroups presented a considerable number of patients reporting a physical and/or psychological trauma at the onset of the skin disease and/or at relapses (Table 2). Furthermore, HAM-D, HAM-A, SCL-90-R, and GSI total score means differed remarkably in patients of the severe subgroup compared to those of the other subgroups (Table 2). Similarly, the HAM-D and HAM-A total score mean of the marked subgroup patients differed significantly compared to those of the minimal subgroup (*p* < 0.001).

A family and/or personal psychiatric history emerged solely in the patients belonging to marked and severe subgroups. Specifically, 5/26 patients with positive familial psychiatric history pertained to the marked subgroup and 11/64 to the severe, 3/26 with a personal psychiatric history pertained to the marked subgroup, and 5/64 to the severe.

### 3.3. Analysis of Criteria for Diagnosing Depression in Psoriasis Patients

When we considered the HAM-D depressed mood item score (Table 3), the result was that a large number of psoriasis patients of the severe subgroup (48.3%) were diagnosed with depression (*p* < 0.05). Differently, a smaller prevalence rate (19.2%) emerged when we used the HAM-D total score. Indeed, 23 (19.2%) patients had a moderate/severe depression (HAM-D score ≥ 18), 26 (21.6%) a mild depression (HAM-D score 8–17), and 15 (12.5%) no depression (HAM-D score < 7). As reported in Table 3, the number of patients of the severe subgroup showed a HAM-D total score higher compared to the other subgroups (*p* < 0.005) (Table 3).

Moreover, when we used the severity index of psychopathological distress as a discriminant factor (GSI ≥ 70), we identified most cases of depressed patients in the severe subgroup (31.7%) (*p* < 0.0001) (Table 3).

Finally, DSM-IV-TR criteria enabled us to diagnose different mood disorders. The data analysis evidenced that only 16.7% of psoriasis patients of the severe subgroup had a diagnosis of major depressive disorder (MDD), 4.2% a depressive disorder not otherwise specified (DD-NOS), and 14.2% an adjustment disorder with depressed mood (AjDDM) (Table 3). All patients with an MDD diagnosis were assigned to the severe subgroup, except for one patient assigned to the marked subgroup. DD-NOS and AjDDM diagnoses, instead, were present in all the five subgroups (Table 3).

### 3.4. Coping Strategies and Correlations with Psychopathological Variables

The analysis of data collected using the COPE Inventory evidenced no significant differences concerning coping strategies in the five subgroups of our cohort of psoriasis patients. Correlation analysis, instead, evidenced that behavioral disengagement was the sole dysfunctional coping strategy adopted by patients of all subgroups. Furthermore, such strategy positively correlated with all the criteria we used to diagnose depression or depressed mood in the severe subgroup and negatively with the SF-36 Mental Health subscale (MSC), as illustrated in Table 4.

Another dysfunctional coping strategy adopted in the severe subgroup was substance use, which positively correlated only with HAM-A total score (*p* < 0.05), SCL-90-R depression subscale (*p* < 0.05), and GSI total score (*p* < 0.001). Further analyses evidenced denial as a further dysfunctional coping strategy used solely by psoriasis patients of the minimal subgroup and positively correlated only with HAM-D depressed mood subscale score (*p* < 0.001).

## 4. Discussion

In our study on a psoriasis outpatients cohort, we reported that the degree of the comorbid depression severity occurred in parallel with that of the skin disease. Indeed, when we subgrouped our cohort according to psoriasis severity criteria, we evidenced that most patients of the severe psoriasis subgroup (PSOdisk total score > 50) had a diagnosis of moderate/severe depression. In addition, we highlighted that the prevalence of comorbid depression proved to be different when multivariate diagnostic criteria were used to assess the mood disorder or the sole depressive mood symptom. Correlation analysis of the criteria we used to diagnose depression or depressed mood indicated that a dysfunctional coping strategy was highly and positively correlated only in patients of the severe subgroup. Such an outcome might suggest that dysfunctional coping strategies increase the depression burden. Differently, a negative correlation emerged between the SF-36 MCS and behavioral disengagement, suggesting that a worse mental health-related QoL, due to psychopathological distress, might induce patients with a marked/severe psoriasis to prefer dysfunctional coping strategies.

The prevalence of comorbid depression in psoriasis patients is reported to vary considerably due to study design or population, sample size, assessment tools, and cut-off values for psychopathological scales [39,40,41,42]. In addition, the diagnosis and severity of the mood disorder may appear different depending on whether psychopathological questionnaires are self-administered by the patients or completed by the physicians after the clinical interview. Furthermore, dermatologists might provide biased prevalence estimates of comorbid depression due to limited familiarity with the various mental health diagnostic criteria and assessment tools.

Depression in psoriasis patients is a challenging issue concerning detection and correct diagnosis. Most studies are based on validated questionnaires and dermatologists’ assessments of the basic depressive symptoms such as the General Health Questionnaire (GHQ) and the Hospital Anxiety and Depression Scale (HADS) [42,43]. Other studies used diagnostic criteria of both International Classification of Diseases (ICD) and DSM classification of psychiatric diseases. By using such tools, the percentage of patients with depression proved to be lower, precisely 12% by ICD-10 [43] and 19% by the DSM-IV [44], as occurred in our cohort when we applied the DSM-IV-TR criteria (Table 3). Saito et al. [45] reported that the ICD-10 proved to be more efficient to identify the mild range of depressive symptoms, while the DSM-IV the moderate-severe range. The DSM-IV diagnosis requires experienced psychiatrists or psychologists, in addition to a considerable amount of time needed to use the multiple assessment tools. Consequently, DSM-IV is scarcely adopted in a dermatology setting.

Only a few studies investigated depression in psoriasis patients through the application of DSM-IV-TR criteria, which require a minimum of five symptoms among which either depressed mood or anhedonia (loss of interest or pleasure) perduring over two weeks, as occurred in our study. Also, besides all the depressive symptoms, we considered the “Gestalt” of depressive patients during the diagnostic process [46], an issue long neglected in the literature [47]. The face-to-face psychiatric interview, indeed, remains crucial for achieving an accurate depression diagnosis.

For the first time in the literature, we performed a comprehensive analysis of depression prevalence in outpatients subgrouped by psoriasis severity. We also illustrated how results varied according to the diagnostic criteria used (Table 3). Only the severe psoriasis subgroup compared with the other subgroups showed a higher percentage of patients with depressed mood and a moderate/severe type of depression diagnosis. Furthermore, in line with what has been previously reported [3], women with psoriasis are at a higher risk than men of suffering from comorbid depression. Indeed, the severe subgroup of our cohort included a slightly higher but significant number of female patients (*p* < 0.0001) (Table 1). Furthermore, the severe subgroup obtained higher HAM-D, and HAM-A mean total scores and a degree of psychological distress greater than 0.70, which is the cut-off value to discriminate relevant psychopathological distress (*p* < 0.0001) (Table 3). Thus, the first element to stress is that the severity of the skin disease certainly influenced patients’ psychopathological status. Our overall clinical judgment was that the severity of psoriasis occurred in parallel with depressive symptoms, thus indicating a possible correlation between psoriasis and depression [48]. In fact, psoriasis and depression are reported to share biological mechanisms such as the high levels of proinflammatory cytokines interleukin (IL)-1 and IL-6, thus showing that an inflammatory process might be involved in both diseases progressing [49,50].

According to the DSM-IV-TR criteria, the severe subgroup displayed also the presence of a few patients diagnosed with DD-NOS and AjDDM. Interestingly, most patients diagnosed with DD-NOS and AjDDM (17/22) presented psychological traumas at onset and/or at relapses of the skin disease. Instead, only a few (6/22) had a personal psychiatric history. Additionally, 50% of the AjDDM patients were physically compromised, overweight or obese (11/16), with high PSOdisk subscale scores of health, itch, and skin involvement in addition to arthropathic psoriasis diagnosis. Schmid-Ott et al. [51] reported that the negative effects of psoriasis might concern two different levels: physical and mental. Accordingly, our AjDDM psoriasis patients might appear to counteract their perceived high psychological distress (GSI mean score 1.12 ± 0.42) with higher severity of somatic symptoms. Differently, MDD patients showed greater and prevalent impairment of the mental status level [8]. Itch (*p* < 0.0001), shame (*p* < 0.0001), and skin involvement (severe vs. minimal *p* < 0.0001, vs. mild *p* < 0.001, vs. moderate *p* < 0.009, and vs. marked *p* < 0.05) PSOdisk subscale scores also significantly increased in the severe subgroup patients. Presumably, such symptoms in patients with an MDD diagnosis contributed to increase the psychological burden rather than the physical severity [52,53].

Instead, in other psoriasis patients, psychological impact and physical severity might be unrelated, due to functional coping strategies used to manage the disease-related distress and also to positive external factors, such as social support, mitigating the burden of disease [54,55,56,57]. Other factors may intervene in such complex system adopted by patients to deal with the stress due to psoriasis and comorbid depression, for instance, a decreased resilience to respond to stress to achieve goals at a minimal psychological and physical cost [58], and personality traits as patient’s patterns of thoughts, feelings, and behaviors [59].

A few studies suggest that the relationship between psychological distress and the depression burden may cause maladaptive cognitive schemas due to somatic symptoms and negative emotions [54,56]. Our results, derived from the correlation analysis between coping strategies and depression diagnostic criteria, indicated that behavioral disengagement was highly and positively correlated with all the diagnostic criteria only in patients of the severe subgroup (Table 4). Another negative correlation emerged by using the SF-36 Mental Health subscale. The outcome illustrated that severe psoriasis patients with comorbid depression adopted more frequently behavioral disengagement, liable to worsen their QoL. Consequently, in our severe psoriasis patients, a relationship might exist between behavioral disengagement and the severity of psychological distress due to depression. Instead, patients affected by other chronic disorders managed the disease-related psychological distress by using coping strategies other than behavioral disengagement [57,58,60,61].

In light of our results, we recommend that dermatologic and psychiatric follow-up of psoriasis patients might jointly contribute to modulating psychopathological processes such as interpersonal functioning, emotion regulation, coping with stress, and mental health. In particular, psychological intervention may enable patients to overcome attachment insecurity by developing a stable mental foundation, increasing resilience in coping with stressful life events, and avoiding collapsing psychologically during a crisis.

Finally, we need to mention a few limitations pertinent to our study. Firstly, the relatively limited number of outpatients and their heterogeneity concerning psoriasis symptoms and clinical subtype, preventing our results’ generalizability. Secondly, the temporal frame considered in the psychopathological questionnaires (one or four weeks) and the patients’ self-assessment of general psychopathology, subject to personal perceptual bias. Thirdly, a family or personal psychiatric history of psoriasis patients in the marked and severe subgroups, possibly contributing to the higher prevalence of depressive symptoms in both groups. Indeed, we had only 5/26 patients with a positive familial psychiatric history in the marked subgroup and 11/64 in the severe. A total of 3/26 and 5/64 patients, on the other hand, had a positive personal psychiatric history. Fourthly, the results subject to bias for a few clinical data collected retrospectively.

Finally, all patients were on topical and/or systemic therapy at enrollment time and surveyed only once before starting a biological treatment.

## 5. Conclusions

Depression is frequently associated with psoriasis and manifests with varying degrees of severity. Identifying such mental disease is a complex issue, mainly when using various assessment tools. We reported different specificity, frequency, and severity of depression based on data resulting from several assessment tools. Standardized DSM-IV-TR criteria showed a lower frequency and a higher specificity of depression diagnosis in our cohort. In addition, DSM-IV-TR displayed other mood disorders, like depressive disorder not otherwise specified (DD-NOS), adjustment disorder with depressed mood (AjDDM), besides major depressive disorder (MDD).

Overall, the particularity of our study is the severe psoriasis subgroup comprising most patients as recognized affected by depression. Consequently, skin disorder severity appears to contribute significantly to the presence of depression and related severity. Furthermore, we emphasized that concurrent psychological distress and inadequate coping strategies influenced the depression outcome, an issue scarcely addressed in the literature.

Problematic early depression diagnosis in psoriasis patients might affect assessing and treating this severe disease, whose delayed or inappropriate treatment may cause suicide risk. We also remarked that a prompt depression diagnosis would allow physicians to provide the most appropriate psychological and/or pharmacological intervention.

Psychological distress, depression, and dysfunctional coping strategies combined with psoriasis require enhanced public health interventions to ameliorate the QoL of patients, especially those at high suicidal risk.

To conclude, cooperation between dermatologists and mental health specialists will be of paramount importance to provide comprehensive care to psoriasis patients, who are to shoulder an enormous burden.

## Figures and Tables

**Table 1:** Sociodemographic and clinical characteristics of psoriasis patients enrolled in the study (N = 120) and subgrouped according to the PSOdisk score category.

	Total Group	Minimal (<9)	Mild (9–15)	Moderate (16–30)	Marked (31–50)	Severe (>50)	*p*-Value
Number of patients/categories	120	18	4	8	26	64	
Gender, male (n)	75	17	2	7	20	29	0.0001
Age, years (mean ± sd)	49.1 ± 12.6	46.0 ± 12.3	61.5 ± 18.7	42.2 ± 17.1	48.1 ± 10.7	50.5 ± 11.9	0.253
Duration of illness, years (mean ± sd)	17.4 ± 12.5	18.2 ± 11.7	17.2 ± 16.6	10.1 ± 7.6	17.4 ± 12.5	18.1 ± 12.9	0.699
Educational level (n):							0.927
- Primary school	27	3	1	1	6	16	
- Middle school	49	8	3	3	10	25	
- High school	35	5	-	4	8	18	
- University	9	2	-	-	2	5	
Marital status (n):							0.803
- Single	18	4	-	3	3	8	
- Married	95	13	4	5	22	51	
- Divorced	4	1	-	-	-	3	
- Widowed	3	-	-	-	1	2	
Occupational status (n):							0.05
- Employed	59	7	1	6	15	30	
- Unemployed	53	11	1	2	9	30	
- Retired	8	-	2	-	2	4	
Socioeconomic condition, (n):							0.679
- Poor	20	3	-	-	4	13	
- Fair	67	8	2	5	15	37	
- Good	33	7	2	3	7	14	
Smoker (yes, n)	66	11	1	4	12	38	0.537
Alcohol use (yes, n)	4	1	-	-	-	3	0.745
Dietary habits, irregular (yes, n)	53	4	1	4	14	30	0.249
Body Mass Index (BMI) (mean ± sd)	29.1 ± 6.3	26.6 ± 3.2	27.7 ± 2.4	28.0 ± 6.4	30.0 ± 4.8	29.7 ± 7.4	0.654

**Table 2:** Psoriasis and psychopathological characteristics of patients enrolled in the study (N = 120) and subgrouped according to the skin disease severity (PSOdisk score).

	Total Group	Minimal (<9)	Mild (9–15)	Moderate (16–30)	Marked (31–50)	Severe (>50)	Statistic
PSOdisk, mean total score (±sd)	49.1 ± 27.9	3.7 ± 2.5	13.7 ± 1.5	24.25 ± 4.5	40.0 ± 5.5	70.9 ± 13.8	* a
PASI, mean total score (±sd)	10.1 ± 3.4	10.3 ± 3.0	9.5 ± 1.0	7.0 ± 3.7	9.5 ± 2.9	10.7 ± 3.6	* b
Arthritis, (yes, n, %)	31	2 (1.7%)	-	3 (2.5%)	8 (6.7%)	18 (15.0%)	** c
Itch, (yes, n) (%)	100	5 (4.2%)	4 (3.3%)	6 (5.0%)	25 (20.8%)	60 (50.0%)	** a
Trauma (yes, n, %):							
- At psoriasis onset	80	6 (5.0%)	3 (2.5%)	5 (4.2%)	18 (15.0%)	48 (40.0%)	** d
- At psoriasis relapse	51	5 (4.2%)	-	4 (3.3%)	8 (6.7%)	34 (28.3%)	** e
Psychiatric family history (yes, n, %)	16	-	-	-	5 (4.2%)	11 (9.2%)	** f
Psychiatric personal history (yes, n, %)	9	0 (0.0%)	1 (0.8%)	0 (0.0%)	3 (2.5%)	5 (4.2%)	** g
HAM-D, mean total score (±sd)	11.7 ± 7.6	7.2 ± 5.4	7.2 ± 6.6	9.5 ± 5.7	8.8 ± 5.2 ^s^	14.8 ± 8.1	* h, l
HAM-A, mean total score (±sd)	12.5 ± 10.6	5.6 ± 6.1	5.5 ± 7.1	6.9 ± 4.2	9.4 ± 11.1 ^s^	16.8 ± 10.3	* m, n
SCL-90-R:							
- Total score (mean ± sd)	59.0 ± 53.1	22.3 ± 22.6	12.2 ± 11.3	28.0 ± 20.3	39.8 ± 34.1	84.0 ± 57.0	* p, q, r
- GSI (mean ± sd)	0.65 ± 0.59	0.25 ± 0.25	0.14 ± 0.12	0.31 ± 0.22	0.44 ± 0.37	0.93 ± 0.63	* m, p, e

* One-way ANOVA with post hoc Bonferroni test; ** chi-square test. a *p* < 0.0001 severe vs. all other subgroups; b *p* < 0.05 severe vs. moderate; c *p* < 0.129; d *p* < 0.302; e *p* < 0.881; f *p* < 1.0; g *p* < 0.343; h *p* < 0.001 severe vs. minimal; l *p* < 0.005 severe vs. marked; m *p* < 0.0001 severe vs. minimal; n *p* < 0.05 severe vs. marked; *p* < 0.0001 severe vs. minimal; q *p* < 0.05 severe vs. mild and moderate; r *p* < 0.001 severe vs. marked; s *p* < 0.001 vs. minimal.

**Table 3:** Data indicating a depression diagnosis based on different assessment criteria in our cohort (N = 120) of psoriasis patients subgrouped by the severity of skin disease (PSOdisk score category).

PSOdisk Category	Minimal (<9)	Mild (9–15)	Moderate (16–30)	Marked (31–50)	Severe (>50)	*p*-Value
Total patients (N, %)	18 (15.0%)	4 (3.3%)	8 (6.6%)	26 (21.6%)	64 (53.3%)	
Diagnostic criteria:						
HAM-D Depressed mood item (n, %)	9 (7.5%)	2 (1.7%)	5 (4.2%)	19 (15.8%)	58 (48.3%)	0.05 *
HAM-D total score (n, %):						0.005 *
≤7	12 (10.0%)	3 (2.5%)	4 (3.3%)	12 (10.0%)	15 (12.5%)	
8–17	5 (4.2%)	1 (0.8%)	3 (2.5%)	12 (10.0%)	26 (21.6%)	
≥18	1 (0.8%)	-	1 (0.8%)	2 (1.7%)	23 (19.2%)	
GSI ≥ 0.70 (n, %)	1 (0.8%)	-	1 (0.8%)	7 (5.8%)	38 (31.7%)	0.0001 **
DSM-IV-TR criteria (n, %):						0.001 *
MDD	-	-	-	1 (0.8%)	20 (16.7%)	
DD-NOS	-	1 (0.8%)	1 (0.8%)	2 (1.7%)	5 (4.2%)	
AjDDM	1 (0.8%)	-	1 (0.8%)	5 (4.2%)	17 (14.2%)	

MDD = Major depressive disorder; DD-NOS = Depressive disorder not otherwise specified; AjDDM = Adjustment disorder with depressed mood. * Chi-square test; ** one-way ANOVA with post hoc Bonferroni test.

**Table 4:** Correlation analysis between the behavioral disengagement COPE subscale, psychopathological variables (HAM-D depressed mood and SCL-90-R Depression subscale, HAM-D, HAM-A, and GSI total score), and the SF-36 Mental Component Summary (MCS) in our cohort of psoriasis patients (N = 120) subgrouped by the severity of skin disease.

Behavioral Disengagement										
PSOdisk Category	Minimal		Mild		Moderate		Marked		Severe	
	r	*p*	r	*p*	r	*p*	r	*p*	r	*p*
HAM-D depressed mood subscale	0.430	0.214	0.000	1.000	0.578	0.133	0.223	0.273	0.411	**0.001**
HAM-D total score	0.699	0.025	0.743	0.410	−0.087	0.838	0.519	**0.007**	0.279	**0.027**
HAM-A total score	0.192	0.595	0.726	0.273	0.005	0.991	0.285	0.157	0.358	**0.004**
SCL-90-R depression subscale	0.207	0.565	0.904	0.095	0.057	0.893	0.397	**0.04**	0.467	**0.0001**
GSI total score	0.202	0.575	0.943	0.057	0.081	0.849	0.519	**0.007**	0.410	**0.001**
SF-36 MCS	0.370	0.293	0.254	0.746	−0.225	0.592	−0.589	**0.002**	−0.427	**0.0001**

Bold typeface indicates statistical significance level of *p* values.

## Data Availability

Data supporting reported results can be found in clinical records of Dermatology Unit, Department of Mental and Physical Health and Preventive Medicine, University of Campania “Luigi Vanvitelli”.
